# Postinfectious Cerebellar Syndrome With Paraneoplastic Antibodies: An Association or Coincidence?

**DOI:** 10.7759/cureus.10190

**Published:** 2020-09-01

**Authors:** Sajid Hameed, Mukesh Kumar, Pal Satyajit Singh Athwal, Sukhmanii Kahlon, Nimit Dalal

**Affiliations:** 1 Neurology, Aga Khan University, Karachi, PAK; 2 Neurology, Shaheed Mohtarma Benazir Bhutto Medical University, Larkana, PAK; 3 Internal Medicine, Saraswathi Institute of Medical Sciences, Hapur, IND; 4 Internal Medicine, Medical University of the Americas, Camps, KNA; 5 Internal Medicine, Trumbull Regional Medical Center, Warren, USA

**Keywords:** acute cerebellitis, postinfectious cerebellitis, paraneoplastic cerebellitis, anti-ri antibodies, anti-amphiphysin antibodies

## Abstract

Cerebellar ataxia has a very broad differential diagnosis in adults, including paraneoplastic and postinfectious etiologies. We report a case of a 56-year-old male presented with right-sided cerebellar dysfunction preceded by fever and headache. He was diagnosed with subacute postinfectious cerebellar ataxia. Blood serology showed the presence of anti-amphiphysin and anti-Ri (ANNA-2, antineuronal nuclear autoantibody type 2) antibodies, which have a known association with cerebellar syndrome. The patient subsequently improved with the steroids. Although no evidence of an underlying tumor was found in the patient, the presence of the paraneoplastic antibodies remains a mystery. We suggest a probable association of these antibodies with the postinfectious cerebellar syndrome.

## Introduction

A cerebellar syndrome varies from the classical signs and symptoms of ataxia, dysmetria, and dysdiadochokinesia to life-threatening herniations [1]. A wide range of disorders involve the cerebellum, including metabolic, vascular, neoplastic, inflammatory, and infectious etiologies. However, isolated cerebellar involvement is mostly infectious or postinfectious [2]. Differential diagnosis includes viral cerebellitis, postinfectious cerebellitis, autoimmune cerebellitis, paraneoplastic cerebellitis, cerebellar space-occupying lesions, drug intoxication, vitamin deficiency (thiamine, vitamin E, vitamin B12), meningoencephalitis, cerebellar stroke, and Creutzfeldt-Jakob disease.

Diagnosis is based on the clinical findings; however, MRI, cerebrospinal fluid (CSF), and laboratory findings are used to support the diagnosis and confirm the etiology. Treatment depends on the underlying etiology. We report a case of a subacute cerebellar syndrome in a 54-year-old male patient whose presentation and CSF findings are suggestive of postviral etiology but are also positive for anti-amphiphysin and anti-Ri antibodies. These antibodies are found in neurological paraneoplastic syndromes (NPS), which are the autoimmune response in less than 1% of cancer patients [3].

## Case presentation

**Figure 1:**
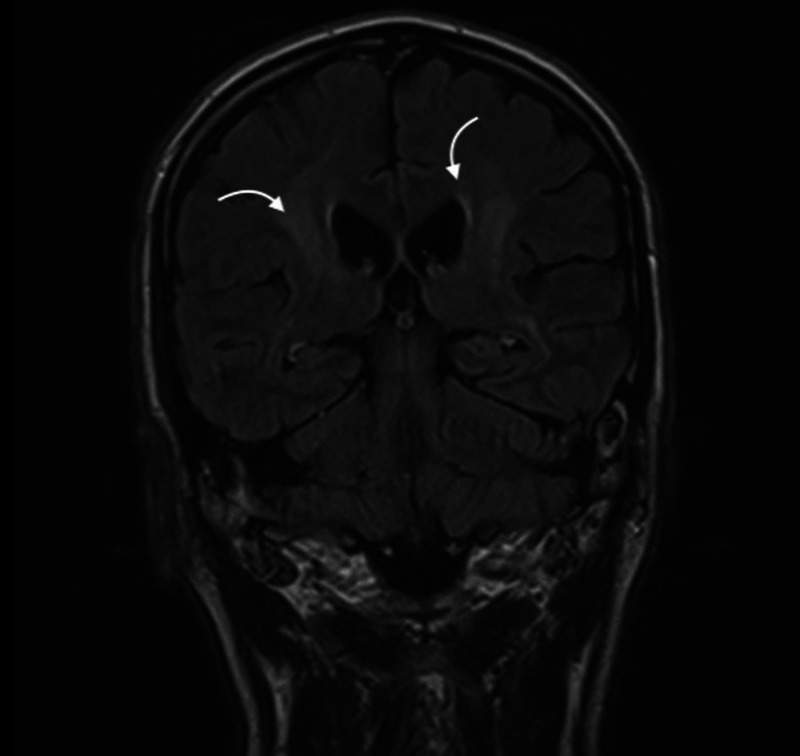
MRI demonstrating abnormal non-specific T2 hyperintense and mild FLAIR hyperintense signals. FLAIR, fluid-attenuated inversion recovery

A 54-year-old male presented with complaints of headache, fever for the last six weeks, and difficulty walking for the last three weeks. Headache was initially moderate in intensity, generalized but more at the occipital region associated with nausea and vomiting. Fever was low grade without any associated symptoms. The patient was also having difficulty maintaining balance with a history of repetitive falls during walking. Past medical history is unremarkable. He has a history of 15 pack-year of cigarette smoking. He denies any exposure to toxins and by occupation is a farmer. On physical examination, higher mental functions were intact. Cranial nerve examination was normal. No motor deficit was noted. He had prominent past-pointing and dysdiadochokinesia on the right side. He was constantly swaying towards the right side on standing and was unable to walk. A provisional diagnosis of the right-sided cerebellar syndrome was made. The laboratory investigations were within normal limits except positive IgG antibodies for Cytomegalovirus. Cranial MRI revealed diffuse abnormal non-specific T2 and fluid-attenuated inversion recovery (FLAIR) hyperintense signals, involving the bilateral periventricular deep white matter, centrum semiovale, and both thalami (Figure 1).

The CSF examination showed a white blood cell count of 36 × 10^3 ^cells/µL (normal <5 × 10^3 ^cells/µL) with 90% of lymphocytes and a protein of 97 mg/dL (normal <40 mg/dL). A polymerase chain reaction (PCR)-based BioFire® meningitis panel (BioFire Diagnostics, Salt Lake City, UT) for some known pathogens came out to be negative (Table 1). Blood and CSF cultures did not grow any organism. 

**Table 1:** Cerebrospinal Fluid Findings

Organisms	Results
Escherichia coli K1	Not detected
Haemophilus influenzae	Not detected
Listeria monocytogenes	Not detected
Neisseria meningitidis	Not detected
Streptococcus agalactiae	Not detected
Streptococcus pneumoniae	Not detected
Cytomegalovirus	Not detected
Enterovirus	Not detected
Herpes simplex virus 1 and 2	Not detected
Human herpesvirus 6	Not detected
Human parechovirus	Not detected
Varicella zoster virus	Not detected
Cryptococcus neoformans/gattii	Not detected

Lab work for some autoantibodies was ordered, and results are shown in Table 2. Anti-amphiphysin and anti-Ri antibodies were positive.

**Table 2:** Paraneoplastic Antibody Panel

Antigen antibodies	Results
Amphiphysin (Amp)	Positive
CV2	Negative
PNMA2/Ta	Negative
Ri	Positive
Yo	Negative
Hu	Negative
Recoverin	Negative
SOX1	Negative
Titin	Negative
Control	Negative
Label	Negative

Suspecting some underlying tumor that might be responsible for a cerebellar dysfunction CT scan with contrast of chest, abdomen, and pelvis was performed, which was unremarkable. The patient was started on oral steroids and the condition of the patient markedly improved. The patient was no longer having any problem and examination was normal after a week.

## Discussion

Isolated cerebellar involvement is a rare condition in adults with a wide range of etiologies and outcomes. Pathophysiology depends on the underlying cause of the cerebellum dysfunction. In a literature review about the acute cerebellitis in adults, infectious etiology was seen in 8 out of 35 cases (23%), but the majority of the cases were idiopathic [1]. A parainfectious or a postinfectious cause of subacute cerebellitis is possible in our case because of the preceding history of fever, headache, and the CSF findings. Previous cases of acute cerebellitis associated with Epstein-Barr virus (EBV) have been reported in the literature, but in our case, no antibodies against EBV were found [2,4].

Paraneoplastic cerebellar syndrome is a well-known entity. Anti-amphiphysin antibodies are found to be positive in many NPS with an underlying tumor. Antoine et al. described five cases with anti-amphiphysin antibodies and underlying tumors, such as breast cancer, small cell cancer of the lung, and ovarian cancer [3]. In our case, no underlying tumor was found despite extensive imaging. Anti-amphiphysin antibodies were also found to be associated with Stiff-person syndrome [5]. Anti-Ri antibodies are well known for the association with paraneoplastic opsoclonus and other NPS [6]. In our case, both the paraneoplastic antibodies were found without an associated underlying primary tumor. Paraneoplastic antibodies can be detected a few weeks to months before the primary tumor can be identified, but in our case, no tumor was identified on the 12-month follow-up. The presence of the paraneoplastic antibodies in our patient remains a mystery. Further studies are needed to evaluate the frequency of these paraneoplastic antibodies in the normal population as well as their association with parainfectious neurological syndromes.

## Conclusions

Isolated cerebellar syndrome is rare and has a wide range of etiologies. Parainfectious and paraneoplastic etiologies should always be considered. Although amphiphysin antibodies and anti-Ri antibodies are associated with NPS including cerebellar syndrome, their association with parainfectious etiologies is unclear. Further studies are needed to evaluate this association and the importance of the presence of these antibodies in such patients.
